# HIV, STI prevalence and risk behaviours among women selling sex in Lahore, Pakistan

**DOI:** 10.1186/1471-2334-11-119

**Published:** 2011-05-11

**Authors:** Mohsin Saeed Khan, Magnus Unemo, Shakila Zaman, Cecilia Stålsby Lundborg

**Affiliations:** 1 Mohsin Saeed Khan, Division of Global Health (IHCAR), Department of Public Health Sciences, Karolinska Institutet, Stockholm, Sweden; 2 Magnus Unemo, National Reference Laboratory for Pathogenic Neisseria, Department of Laboratory Medicine, Clinical Microbiology, Örebro University Hospital, and School of Health and Medical Sciences, Örebro University, Örebro, Sweden; 3 Shakila Zaman, Institute of Public Health, Lahore, Pakistan; 4 Cecilia Stålsby Lundborg, Division of Global Health (IHCAR), Department of Public Health Sciences, Karolinska Institutet, Stockholm, Sweden

## Abstract

**Background:**

More than 340 million cases of curable sexually transmitted infections (STIs) were estimated to have occurred worldwide in 1995. Previous studies have shown that the presence of other concomitant STIs increases the likelihood of HIV transmission. The first national study of STIs conducted in Pakistan in 2004 revealed a high burden of STIs among women selling sex. The HIV epidemic in Pakistan has thus far followed the "Asian epidemic model". Earlier studies among women selling sex have shown a low prevalence of HIV coupled with a low level of knowledge about AIDS. The aim of our study was to estimate the prevalence of HIV and STIs, and assess knowledge and risk behaviours related to HIV/STI, among women selling sex in Lahore, Pakistan.

**Methods:**

A total of 730 participants were recruited through respondent-driven sampling. The participants were women selling sex in three areas (referred to as "A", "B", and "C") of Lahore. A structured questionnaire addressing demographic information, sexual life history, sexual contacts, and knowledge and practices related to HIV/STI prevention was administered by face-to-face interview. Biological samples were obtained from all participants and tested for HIV, *Treponema pallidum*, *Neisseria gonorrhoeae*, *Chlamydia trachomatis *and *Trichomonas vaginalis*. Pearson's chi-square and multivariable logistic regression analysis were performed to test associations between potential risk factors and specified diagnosed infections.

**Results:**

The prevalence of HIV infection was 0.7%, *T pallidum *4.5%, *N gonorrhoeae *7.5%, *C trachomatis *7.7% and *T vaginalis *5.1%. The participants had been selling sex for a median period of seven years and had a median of three clients per day. Sixty five percent of the participants reported that they "Always use condom". The median fee per sexual contact was Rs. 250 (3 Euro). Compared to Areas A and C, women selling sex in Area B had a significantly higher risk of chlamydial infection, gonorrhoea and trichomoniasis. Among the participants, 37% had correct knowledge about HIV/AIDS transmission and its prevention.

**Conclusions:**

The prevalence of HIV was <1%, and of any other STI 18.5% among participating women selling sex in Lahore, Pakistan. A reasonably high condom use, a relatively low number of sexual partners, and a relatively low prevalence of STIs might have contributed to the low HIV prevalence.

## Background

More than 340 million cases of curable sexually transmitted infections (STIs) including *Treponema pallidum, Neisseria gonorrhoeae, Chlamydia trachomatis *and *Trichomonas vaginalis *were estimated to have occurred worldwide in 1995 [1]. Previous studies have shown that the presence of other concomitant STIs increases the likelihood of HIV transmission [2-4].

In Pakistan, the estimated number of people living with HIV (PLHIV) was 96,000 in 2008, with an adult HIV prevalence estimated at 0.1% [5]. The HIV epidemic in Pakistan has thus far followed the so-called Asian epidemic model, where HIV initially affects injecting drug users (IDUs) before passing on to the general population through the bridging populations of men who have sex with men (MSM) and/or women selling sex [6]. There is also evidence of non-concomitant sexual networking among IDUs, women selling sex and MSMs. The 2^nd ^Generation HIV Surveillance, conducted in 2006-07, revealed that 9.9% of women selling sex reported having sex with IDUs, and 22% of IDUs reported having bought sex from women selling sex [7].

The first national STI study in Pakistan was conducted in 2004 in order to determine the disease burden of HIV and STIs. The study showed a high burden of STIs among women selling sex, with the following prevalences; *T. pallidum *7%, *N. gonorrhoeae *12%, *C. trachomatis *11% and *T. vaginalis *19%. The study also highlighted a low level of knowledge about AIDS among the women selling sex; 24% had never heard of AIDS and 26% had not used a condom with their most recent sexual partner (http://www.nacp.gov.pk/library/reports/). In Pakistan, the terms HIV and AIDS are commonly used synonymously. The Ministry of Health, Government of Pakistan, through the National and Provincial AIDS Control Program introduced preventive interventions among the most-at-risk populations in various urban settings in 2005 through public private partnerships. These interventions were focused on provision of curative and preventive services, including syndromic management of STIs, provision of condoms, and behaviour change communication. The interventions were also tied with the 2^nd ^generation HIV surveillance among most-at-risk population, which estimated the HIV prevalence and its related risk behaviours, but did not measure the prevalence of STIs (http://nacp.gov.pk). Our study assessed both the HIV and STI prevalence with related risk behaviours.

The history and context of sex work among women selling sex in Lahore, Pakistan has been described earlier [8,9]. Briefly, the Mughal Dynasty, which ruled most of India, Pakistan, Bangladesh, and Afghanistan for over four centuries patronized artists, singers, and dancers by establishing settlements near the palaces where they lived. One such area was established next to the Royal Fort of Lahore. This area in Lahore is still called Heera Mandi (the market of diamonds) or Bazaar-e-Husn (the market of beauty), and is the oldest establishment in Pakistan where women sell sex [9]. Music, singing, dancing, and selling sex was occurring side by side in these areas. Traditionally, the women passed on their profession, including selling sex to their daughters, but not to their daughters-in-law. The latest estimated figure of women selling sex in Lahore is 26,000, which is the highest number of mapped women selling sex in any city in Pakistan (World Bank, 2006). Currently, the women selling sex in Lahore are found to work in at least four different settings or contexts: Brothel based, Kothikhana based, Street based and Home based. In the *Brothel based *typology, Brothels are fixed venues, which are usually operated by "madams" sometimes together with other network operators. Women selling sex live in the house, which is licensed for singing and dancing. These houses are normally located in larger sex work or red light areas and are stable locations, known by clients. Sex work takes place either at the brothel or at the client's residence and the women selling sex have usually no other work. *Kothikhana *is an idiomatic expression for a sex work venue that literally means "grand house". However, Kothikhanas are generally small premises, which are rented by a "madam". They are often located in residential areas and their location is usually kept a secret. A key feature is that they move from time to time when the "madam" determines that the location is unsafe or unsuitable. Women selling sex at Kothikhanas work on weekdays from 9 AM to 5 PM and are hired on fixed rate per client by the "madam". In the *Street based *typology, women selling sex solicit clients in public places such as busy streets, intersections, bus and train stations and marketplaces. Sexual transactions then occur at a venue chosen by the woman selling sex or the client. In the *Home-based *typology, women selling sex usually live with their families. Contact with clients is established by means of mobile phones or through network operators. Sex work takes place either in the client's home or hotels, or a place provided by the network operator. Sex workers usually work part time, operating when required for financial purposes [7].

The aim of our study was to estimate the prevalence of HIV and STIs and assess knowledge and risk behaviours related to HIV/STI among women selling sex in Lahore, Pakistan.

## Methods

### Study design, study population and inclusion by respondent-driven sampling (RDS)

This was a cross-sectional, community-based, quantitative study conducted among women selling sex in Lahore, Pakistan. We defined a woman selling sex as "A female engaged in selling sex, part time or full time, as a means of making a living".

The sample size was calculated by using the computer software EPI info version 6.04D, on the basis of an expected frequency of sexually transmitted infection of 12% ± 3 percent units, and a confidence interval of 95%, resulting in a total required sample size of 726.

The participants (n = 730) were recruited by respondent-driven sampling (RDS) from three areas, mapped in 2004, where they resided as well as sold sex (http://nacp.gov.pk). RDS involves chain referral sampling in a manner that allows it to be qualified as a probability sampling method. RDS has several advantages in recruiting hard-to-access populations, such as broad inclusion, larger recruitment chains, time efficiency and low cost. RDS also statistically adjusts for the biases inherent in how individuals of similar characteristics are networked and are likely to know and recruit each other. Furthermore, RDS works on networks of participants and each wave of recruitment adjusts for inclusion probability [10-13].

Due to the sensitivity of the issues discussed and the possibility of increasing the discrimination against women selling sex, the investigated areas are referred to as "Area A", "Area B" and "Area C". Briefly regarding the RDS, one woman selling sex from each area was randomly selected as a seed. She was given three coupons to bring in three more women selling sex, who were neither pregnant nor menstruating at the time of recruitment. This created what is defined as wave one. The three women recruited through the seeds were given three coupons each to recruit three more women selling sex. This continued until wave five. The chains were followed carefully to ensure that the same woman did not return and also that all recruited women were engaged in selling sex. A confirmation of this was provided by the staff of the clinic who had been working in the areas with women selling sex for the last 4 years and by noting personal identification marks. Our study was conducted among "Home-based" and "Kothikhana-based" women selling sex [7]. In "Area A", women selling sex were "Home-Based". In areas "B" and "C", women selling sex were "Kothikhana"-based. The survey participants received a free clinical examination, HIV and STI testing, and a double financial incentive (for participating as well as bringing in three more women) to compensate for their time and travelling costs.

### Data collection

Data were collected from September to November 2007. A questionnaire with mainly closed-ended questions was developed, which was largely based on an earlier qualitative study conducted in the same areas [8]. The questionnaire, administered by face to face interviews, addressed demographic information, sexual life history, sexual contacts, HIV/STI prevention knowledge and practices. Knowledge was defined as "correct" if the participants could cite two correct modes of transmission, one of which had to be "sexual", and two methods of prevention of HIV/AIDS, one of which had to be "use of condoms" (http://nacp.gov.pk).

The questionnaire was developed in English, translated into Urdu and pre-tested. After revision, the questionnaire was tested again, and further revised. The data collection team was trained in data collection techniques including standardized clinical examination, biological specimen collection, labelling, storage, transportation and how to conduct the face-to face interview using the questionnaire. Clinical examination and sampling of biological specimens were performed using the guidelines of the National AIDS Control Program of Pakistan (http://nacp.gov.pk) and following the phlebotomy procedures as defined by the College of American Pathologists [14].

### Laboratory diagnostics

Serological screening for HIV and *T pallidum *was performed using AxSYM HIV 1/2 gO kit (Abbott Laboratories, Wiesbaden, Germany) and Immunotrep RPR as a qualitative test (Omega Diagnostics Limited, Alva, Scotland, UK), respectively, in accordance with the instructions of the manufacturer. Screening positive HIV and *T pallidum *samples were confirmed using Capillus HIV 1/HIV 2 (Trinity Biotech, Wicklow, Ireland) and TPHA (Randex, Antrim, UK), respectively. For diagnosis of *N gonorrhoeae *and *C trachomatis*, endocervical specimens were analysed using AMPLICOR CT/NG PCR (Roche Diagnostics, Indianapolis, USA), including AMPLICOR *Neisseria gonorrhoeae *Detection Kit and AMPLICOR *Chlamydia trachomatis *Detection Kit, in accordance with the instructions of the manufacturer. For diagnosis of *T vaginalis*, high vaginal swabs were used to collect samples, immediately inoculated into InPouch TV Culture System (BIOMED DIAGNOSTICS, Oregon, USA), incubated and subsequently interpreted in accordance with the instructions of the manufacturer.

### Data analysis

Epi info software version 6.04D was used for data entry. Data were processed and analysed using SPSS 13 (SPSS Inc., Chicago, Illinois, USA). Frequencies, proportions, mean, minimum and maximum were used for the analysis. Pearson's chi-square analysis was performed to test associations between potential risk factors and specified diagnosed infections. Multivariable logistic regression analysis was employed to indicate influencing factors on HIV/STI prevalence. These included age, education, marital status, religion, place of work, condom use, duration of selling sex, number of sexual partners per day, injecting drug use and blood transfusion. In addition RDS Analysis Tool v5.3 (http://www.respondentdrivensampling.org) was used to estimate the transition probability (probability of one group recruiting the other), adjusted mean network size based on the median age, Homophily (a measure of preference for connections to one's own group, which varies between -1 (completely heterophilous) and +1 (completely homophilous), and the confidence intervals.

### Ethics

Study protocols, questionnaires and consent forms were approved by the Ethics Committee of the Pakistan Medical Research Council, Islamabad, Pakistan (2006) and Regional Ethics Committee, Stockholm, Sweden (2006). The participants were informed and gave their consent before inclusion in the study. Participation was linked confidential and participants were given access to their biological results with the appropriate counselling and treatment. HIV positive participants were referred to the Voluntary Counselling and Testing Centre in Lahore for monitoring of the HIV infection, clinical management and social rehabilitation.

## Results

### Study population, risk behaviours and prevalence of HIV and STIs

A total of 730 women, participated in the study. Their reported network consisted of 5,226 women. Among the 730 participants, 66% were Kothikhana-based, and the remainder were home-based. The response rate was 100% among the recruited women, i.e. all the three recruited women by each woman participated. The majority of participants (87%) were Muslims, with the rest being Christians.

The median age of the participants was 30 years (range 13-50 years), of which almost half (47%) were in the age group 30-39 years, 60% had not attended school, and 91% were married.

The transition probability for participants aged 30 and above was 57% within its own group as compared to participants below 30 years of age who were recruiting 50% within themselves. The mean adjusted network size was 3.5 for the former group as compared to the 3.8 for the latter. The value for homophily was 0.022 for the group aged 30 years and above as compared to 0.111 among the participants less than 30 years of age, implying that the participants had homogenous networks. The estimated population proportions with their confidence limits for the age group 30 and above was 0.56 (confidence interval (CI) 95%: 0.52-0.6) and for participants less than age group 30 was 0.43 (CI 95%: 0.39-0.47).

The participants had been selling sex for a median of seven years (range 1-35 years). They reported a median of three clients per day (range 1-12). Seven percent of the participants had female clients. The median fee per sexual contact was Pakistani Rupees (Rs) 250 (3 Euro; range 0.2-61 Euro). Ninety one percent of the participants cited poverty and financial reasons for starting to sell sex, and 5% said that their mother-in-law had forced them into selling sex. Almost all of the participants (99%) said that they continued to sell sex for economic survival. "Always use condoms" was reported by 65% of the participants. Among these, 99% reported using condoms for prevention of pregnancy, 66% for the prevention of HIV and 58% for STI prevention. The area with the lowest reported rate of always using condoms was "Area B" (59%).

Women who reported up to three clients per day were 3.9 times (CI 95%: 2.8-5.4) more likely to also report "Always use condoms" versus women who reported four or more clients per day.

Furthermore, women who reported selling sex for eight years or less were 1.6 times (CI 95%: 1.2-2.3) more likely to "Always use condoms" as compared to women that had been selling sex for nine or more years.

Blood transfusion was reported by 16% of the participants following gynaecological obstetrical surgeries. Among these, 54% claimed that the transfused blood was tested for HIV, hepatitis B and C and malaria. Three participants (0.4%) reported injecting drugs, and one among these reported sharing needles or syringes in a group.

The overall prevalence of HIV and STIs was 0.7% and 18.5% respectively. None of the HIV-positives had a concomitant STI. Among the women who had multiple current non-viral STIs, 1.4% and 3.6% had concomitantly three and two STIs respectively, and 13.6% were infected with one STI. Among the three areas surveyed, women selling sex in Area B had the highest prevalence of HIV (1.2%), *T pallidum *(7.9%)*, N gonorrhoeae *(10.0%), *C trachomatis *(14.9%) and *T vaginalis *(11.2%) (Table 1).

**Table 1:** Background characteristics, risk factors, prevalence of HIV and STIs of participating women selling sex in Lahore, Pakistan

	Place of work No. of participants (%)			Total
	Area "A" n = 248 (34)	Area "B" n = 241 (33)	Area "C" n = 241 (33)	n = 730 (100)
***Age (years)***				
13-19	14 (6)	8 (3)	6 (3)	28 (4)
20-29	116 (47)	103 (43)	89 (37)	308 (42)
30-39	113 (45)	125 (52)	102 (42)	340 (47)
40+	6 (2)	4 (1)	44 (18)	54 (7)
***Educational status (schooling in years)***				
No school	159 (64)	129 (54)	147 (61)	435 (60)
1-5	44 (18)	47 (19)	36 (15)	127 (17)
6-12	45 (18)	65 (27)	58 (24)	168 (23)
***Marital status***				
Single	5 (2)	12 (5)	3 (1)	20 (3)
Married	219 (88)	211 (88)	236 (98)	666 (91)
Divorced	4 (2)	12 (5)	1 (0.5)	17 (2)
Widowed	20 (8)	6 (2)	1 (0.5)	27 (4)
**Risk factors**				
***Condom use***				
Always	181 (73)	143 (59)	147 (60)	471 (65)
Not always	67 (27)	98 (41)	94 (40)	259 (36)
***Duration of selling sex (years)***				
1-5	151 (61)	122 (51)	127 (53)	400 (55)
6-10	50 (20)	43 (18)	44 (18)	137 (19)
11-15	30 (12)	30 (12)	30 (12)	90 (12)
16-20	10 (4)	26 (11)	30 (12)	66 (9)
21-25	7 (3)	11 (5)	9 (4)	27 (4)
26-35	0 (0)	9 (3)	1 (0.4)	10 (1)
***Injecting drugs***				
Yes	2 (0.8)	1 (0.4)	0 (0)	3 (0.4)
No	246 (99)	240 (99)	241 (100)	727 (99)
***Blood transfusion***				
Yes	33 (13)	43 (18)	40 (17)	116 (16)
No	215 (87)	198 (82)	201 (83)	614 (84)
**Prevalence of HIV and STIs**				
HIV	2 (0.8)	3 (1.2)	0 (0.0)	5 (0.7)
*Treponema pallidum*	10 (4.0)	19 (7.9)	4 (1.7)	33 (4.5)
*Neisseria gonorrhoeae*	24 (9.7)	24 (10.0)	7 (2.9)	55 (7.5)
*Chlamydia trachomatis*	9 (3.6)	36 (14.9)	11 (4.6)	56 (7.7)
*Trichomonas vaginalis*	3 (1.2)	27 (11.2)	7 (2.9)	37 (5.1)
***STIs***				
3 concomitant STIs	1 (0.4)	9 (3.7)	0 (0.0)	10 (1.4)
2 concomitant STIs	4 (1.6)	19 (7.9)	3 (1.2)	26 (3.6)
1 STI	35 (14.1)	41 (17.0)	23 (9.5)	99 (13.6)

Potential risk factors for being infected with HIV and/or STIs were also statistically analysed. A significant association was found only for *C trachomatis*, *N gonorrhoeae *and *T vaginalis*. The odds of getting infected with *C trachomatis *and *T vaginalis *were 4.1 (CI 95%; 2.3-7.6) and 6.2 (CI 95%; 2.9-14.2) respectively if the women selling sex were working in Area B as compared to working in Area A and C. In addition the odds of getting infected with *C trachomatis *and *N gonorrhoeae *were 2.5 (CI 95%; 1.4-4.3) and 2.3 (CI 95%; 1.3-4.1) respectively if the women selling sex had between 4 to 12 clients per day as compared to the ones who had 1 to 3 clients per day. The odds to be infected with *C trachomatis *and *N gonorrhoeae *were 2.6 (CI 95%; 1.5-4.7) and 2.0 (CI 95% 1.1-3.5) if the women selling sex reported not always using a condom as compared to the ones who reported they always used condoms with their clients (Table 2).

**Table 2:** STIs and associated risk factors of participating women selling sex in Lahore, Pakistan

STI	Factors		Number (%)	OR (CI 95%)	P value
*Chlamydia trachomatis*	Place of Work	Area A and C	20 (4.1)		
		Area B	36 (14.9)	4.1 (2.3-7.6)	<0.001
	Clients	1-3/day	26 (5.4)		
		4-12/day	30 (12.2)	2.5 (1.4-4.3)	0.001
	Condom use	Always	24 (5.1)		
		Not always	32 (12.4)	2.6 (1.5-4.7)	<0.001
*Neisseria gonorrhoeae*	Clients	1-3/day	26 (5.4)		
		4-12/day	29 (11.8)	2.3 (1.3-4.1)	0.002
	Condom use	Always	27 (5.7)		
		Not always	28 (10.8)	2.0 (1.1-3.5)	0.01
*Trichomonas vaginalis*	Place of work	Area A and C	10 (2.0)		
		Area B	27 (11.8)	6.25 (2.9-14.2)	<0.001

In addition, logistic regression was applied for HIV and STIs, and significant risk behaviours. The odds of being infected with *C trachomatis *and *N gonorrhoeae *were 2.1 (CI 95%; 1.1-3.8) and 1.9 (CI 95%; 1.1-3.5), respectively, if women reported not "always" using condoms as compared to if they reported "always" using condoms.

### Knowledge and attitudes regarding HIV and AIDS

Among the participants, 19% (139) and 83% (606) had heard of HIV and AIDS, respectively. Fifty-five percent did not believe that they were at risk of acquiring AIDS. Approximately 37% had correct knowledge about modes of transmission and measures to prevent AIDS.

If infected, 98% said that they would get themselves treated. When asked what they would do if someone they knew got infected with AIDS, 70% said that they would help her to get treatment, although 40% said that they would also avoid her.

## Discussion

Among the 730 women selling sex in Lahore who participated in our study, 91% were married, 0.7% were infected with HIV and an additional 18.5% were suffering from different curable STIs. Only 19% and 83% of the participants were aware of the terms HIV and AIDS, respectively, and only 37% had correct knowledge about transmission and prevention of AIDS.

According to the 2^nd ^generation HIV surveillance report from 12 major cities in Pakistan, the likelihood of the HIV epidemic affecting women selling sex is very high, as, for example 10% to 32% of the women selling sex have sex with IDUs, who have an overall HIV prevalence of 15.8% (CI 95%: 14.7-16.9; range 1-53%) (http://nacp.gov.pk). In another study conducted in two major cities, 24% and 21% of IDUs reported that they had sex with women selling sex [15,16]. However, in studies conducted among women selling sex, the prevalence of HIV infection has been reported to be less than one percent (http://nacp.gov.pk) [15,16]. There might still be a window of opportunity to keep HIV prevalence below 5% among women selling sex, if the scaling up of services, including laboratory diagnostics, syndromic management and periodic presumptive treatment of STIs, and increase in condom use, is achieved. Such interventions in India among women selling sex have demonstrated that the package of services cited above has the potential to prevent 22%-35% of all new HIV infections among women selling sex [17].

An earlier study from 2004 examining women selling sex (n = 400) in Lahore recruited through RDS in only one of the four areas, found a substantially higher prevalence of most STIs, i.e. *N gonorrhoeae *(12.3%), *C trachomatis *(11%), *T vaginalis *(19.3%), and *T pallidum *(7.1%) (http://nacp.gov.pk). However, our study, which was conducted in three different areas of Lahore, and recruited almost the double number of participants, found lower prevalences of STIs as compared to the 2004 study which was done only in one area of Lahore. Our study as well as another study [15] showed the prevalences of different STIs to be less than 10% among women selling sex. A review of 42 studies on STI rates around the world have emphasised that women selling sex tend to have comparatively higher rates of STI than the general population [18]. In Kolkata, India, women selling sex had a high prevalence of STIs, with 34% testing positive for *N gonorrhoeae *and 23% for *T vaginalis *[19]. In a study performed in Vietnam the prevalence of *N gonorrhoeae *and *C trachomatis *were 14.9% and 48.4%, respectively [20].

The comparatively low levels of HIV and STIs in our study are best understood in the context of what is known about the local sexual and social networks and possibly the transmission patterns. Mathematical models of networks of women selling sex suggest that the prevalence of HIV is extremely sensitive to an increase in condom use and number of irregular partners [21,22]. In "Area B", where women selling sex were Kothikhana based, we found the lowest reported rate of "Always using condoms", the highest number of clients per woman, and hence the highest prevalence of HIV. Furthermore, the low prevalences of HIV and STIs in our study and that of Hawkes et al in two other cities of Pakistan[15], as compared with other studies, could perhaps be explained by relatively high condom use, relatively low numbers of sexual partners and availability of antibiotic treatment through syndromic management of STIs.

In our study, the reported rate of "always use condoms" was high (65%) as compared with previous studies that have reported rates of 21%-47% (http://nacp.gov.pk). Furthermore, in the present study, the duration of selling sex and number of clients had an inverse relationship with condom usage. This is in concordance with a previous study conducted in Vietnam, where having five or less clients per day was associated with consistent condom use [20]. Inaccurate self-reporting of risky behaviours is the primary threat to the validity and utility of research among most-at-risk populations. The self-reported risk behaviours are often not correct and hence have implications for designing future strategies and interventions [23-25].

In our study, 91% of the women selling sex were married. To interpret the results, it is important to understand the context of the decision to sell sex as a married woman in Pakistan. Two thirds of Pakistan's population live on less than Euro 1.34 per day [5], with a total fertility rate of 4.1, an inflation rate of 7.7% (including a food inflation rate of 10%), and a high unemployment rate of 12.6%(http://finance.gov.pk). As a result, the struggle for survival has forced a number of married women, some of whom participated in our study, to sell sex. Approximately 5% of our study participants were forced to sell sex by their mother-in-law. This contradicts reports by both Saeed and Brown, which says that girls marrying into a family of women selling sex in Lahore are commonly forbidden to sell sex and kept in separate quarters at home [9,26]. In our study however, the majority of women did not belong to families of women selling sex.

About 7% of our participants had female clients. This is a new finding and calls for further study of what appears to be a new type of social behaviour in Pakistan. To our knowledge, similar findings have not been reported before.

The low level of knowledge about HIV and AIDS in the present study is consistent with the results of the NACP survey conducted in 12 major cities of Pakistan (http://nacp.gov.pk). However, most alarming is that half of the participants did not have the correct knowledge about HIV transmission and prevention. Fifty five percent claimed that they were not vulnerable to AIDS. This might increase their likelihood of engaging in behaviours that make them vulnerable to exposure to HIV/STIs. Although the perception of being safe from HIV infection and AIDS is lower than the 82% reported by Bokhari [16], this is a huge challenge to those working with HIV/AIDS information among women selling sex in Pakistan.

### Methodological considerations

Sampling procedures in HIV surveillance are designed to bring out unbiased estimates of magnitude, severity, trends in HIV infection rates as well as behavioural patterns that are possibly responsible for driving the epidemic [27]. In the present study, the participants were enrolled by using RDS, which facilitated a zero refusal rate. Furthermore, the double incentive system for participating and recruitment possibly led to an absence of any break in the recruitment chain during any wave, resulting in a highly time-efficient process. Previous studies conducted among women selling sex in Pakistan have used various sampling methods including RDS, cluster sampling, systematic random sampling and snowballing simultaneously to recruit women selling sex working under different conditions (http://nacp.gov.pk). Our study followed the principles of RDS irrespective of the typology of the women selling sex in Pakistan. Furthermore, our study had almost double the number of participants than other studies conducted in Pakistan, such as those reported by Hawkes and Bokhari [15,16].

## Conclusion

Both HIV infection and STIs among women selling sex were relatively low in our study, which is possibly due to a high condom use, relatively low numbers of sexual partners and availability of clinical services, including syndromic management. However, there exists a high risk for a concentrated HIV epidemic among women selling sex due to their low level of knowledge about HIV, risk behaviour and sexual practices.

## Competing interests

The authors declare that they have no competing interests.

## Authors' contributions

MSK, CSL and MU were responsible for the conception and design of the study. MSK and SZ were responsible for the data collection. Field supervision was done by SZ. Data analysis and drafting of the manuscript was done by MSK. All authors were involved in revising the manuscript critically for intellectual content. All authors have given their final approval of the submitted manuscript.

## Pre-publication history

The pre-publication history for this paper can be accessed here:

http://www.biomedcentral.com/1471-2334/11/119/prepub
